# Comparative Transcriptome Analysis of Fetal Skin Reveals Key Genes Related to Hair Follicle Morphogenesis in Cashmere Goats

**DOI:** 10.1371/journal.pone.0151118

**Published:** 2016-03-09

**Authors:** Ye Gao, Xiaolong Wang, Hailong Yan, Jie Zeng, Sen Ma, Yiyuan Niu, Guangxian Zhou, Yu Jiang, Yulin Chen

**Affiliations:** 1 College of Animal Science and Technology, Northwest A&F University, Yangling, People’s Republic of China; 2 College of Life Science, Yulin University, Yulin, People’s Republic of China; China Agricultural University, CHINA

## Abstract

Cashmere goat skin contains two types of hair follicles (HF): primary hair follicles (PHF) and secondary hair follicles (SHF). Although multiple genetic determinants associated with HF formation have been identified, the molecules that determine the independent morphogenesis of HF in cashmere goats remain elusive. The growth and development of SHF directly influence the quantity and quality of cashmere production. Here, we report the transcriptome profiling analysis of nine skin samples from cashmere goats using 60- and 120-day-old embryos (E60 and E120, respectively), as well as newborns (NB), through RNA-sequencing (RNA-seq). HF morphological changes indicated that PHF were initiated at E60, with maturation from E120, while differentiation of SHF was identified at E120 until formation of cashmere occurred after birth (NB). The RNA-sequencing analysis generated over 20.6 million clean reads from each mRNA library. The number of differentially expressed genes (DEGs) in E60 vs. E120, E120 vs. NB, and E60 vs. NB were 1,024, 0 and 1,801, respectively, indicating that no significant differences were found at transcriptomic levels between E120 and NB. Key genes including *B4GALT4*, *TNC*, *a-integrin*, and *FGFR1*, were up-regulated and expressed in HF initiation from E60 to E120, while regulatory genes such as *GPRC5D*, *PAD3*, *HOXC13*, *PRR9*, *VSIG8*, *LRRC15*, *LHX2*, *MSX-2*, and *FOX*N1 were up-regulated and expressed in HF keratinisation and hair shaft differentiation from E120 and NB to E60. Several genes belonging to the *KRT* and *KRTAP* gene families were detected throughout the three HF developmental stages. The transcriptional trajectory analyses of all DEGs indicated that immune privilege, glycosaminoglycan biosynthesis, extracellular matrix receptor interaction, and growth factor receptors all played dominant roles in the epithelial-mesenchymal interface and HF formation. We found that the Wnt, transforming growth factor-beta/bone morphogenetic protein, and Notch family members played vital roles in HF differentiation and maturation. The DEGs we found could be attributed to the generation and development of HF, and thus will be critically important for improving the quantity and quality of fleece production in animals for fibres.

## Introduction

Cashmere goats have double coats consisting of non-modulated fine inner hairs or cashmere fibres produced by secondary hair follicles (SHF) and guard hairs produced by primary hair follicles (PHF), which are invaginated into the basement membrane of the skin (epithelial and mesenchymal compartment) [1, 2]. Cashmere is a fine wool cashmere fibre (generally with diameter < 19 μm) that is used to produce soft luxurious apparel. The number and density of SHF, which affect the yield and diameter of the cashmere fibres, determines the value of the cashmere fleece [3]. Given that the generation of HF is established during early fetal life, and fibre characteristics are realised when the follicles are mature, examination of the processes and transcriptional regulatory mechanisms of the skin epithelium and skin appendage morphogenesis is therefore required to achieve maximum cashmere production [4].

HF morphogenesis is considered to occur in the major stages, for example during induction and initiation, differentiation and maturation [5]. The process of follicle morphogenesis has been studied extensively in murine models, but rarely in goats that produce fibres [5, 6]. A study on fetal HF morphogenesis of the Inner Mongolia Cashmere goats demonstrated that the hair placodes are formed at 55–65 days gestation (~E60), the SHF undergo rapid cytodifferentiation at 105–125 days gestation (~E120), and all PHF and some SHF mature at 135 days gestation [7]. HF morphogenesis is therefore a continuum process between 55 and 135 days of fetal life, which can represent the initiation, differentiation and maturation stages of HF.

The regulation of HF morphogenesis involves a series of complex molecular intercommunications between the single-layered epithelium and dermal cell condensate in skin. The epithelial-mesenchymal interface (EMI) during the organogenesis of HF is presumed to influence cell-substrate interactions [8, 9]. However, fewer studies have been carried out in animals whose skin is comprised of two types of HF, such as cashmere goats [10, 11]. The time point of fetal HF morphogenesis and related transcriptional gene expression in cashmere goats remain to be elucidated. A better understanding of the biological characteristics and regulation of HF morphogenesis may provide approaches to enhance the formation of fleece, whereby proper development is critically important for achieving maximum fleece production.

The structure of the HF in mammals is complex [12]. HF development takes place during fetal skin development and is modulated by extra-follicular macro-environmental factors [12–14]. Considering the complexity of the HF, studies of fetal skin have been valuable for fully identifying DEGs that appear to be developmentally regulated. RNA-seq is an unbiased technology approach for data collection [15, 16]. Previous findings on adult cashmere goats and sheep skin transcriptome analyses identified that many genes and pathways may be important for the regulation of HF cycling and coat colour [17–20]. These studies provided some insights into the genes that play versatile roles in the extra-follicular macroenvironment in goats and sheep. However, the extra-follicular macroenvironmental signalling that regulated fetal HF morphogenesis in cashmere goats is still not understood. Herein, a skin transcriptome analysis at three specific developmental stages, 60- and 120-day-old embryos and newborn (E60, E120 and NB, respectively), was conducted to examine the associated transcriptional genes governing the relevant processes. A number of stage-specific DEGs revealed here represent an important resource for identifying the molecular basis of fetal during skin and HF development in cashmere goats.

## Materials and Methods

### Animals and sample collection

Based on previous artificial insemination records (semen was collected from different rams), nine pregnant Shaanbei White cashmere goats (two years old, weighing 30–40 kg) at the same stage of pregnancy were selected from a breeding farm in Yulin, in the Shaanxi Province of China. All animals underwent identical feeding practices in accordance with the goat farm instructions. Six fetuses were delivered from six different females by caesarean at E60 and E120; E60 represented the initiation stage, and E120 represented the development stage. Three fetuses were also killed within two hours of birth, as a NB, from three different ewes; NB represented the PHF maturation stage. Each time point had three replicates. Skin samples from fetuses were collected from the right mid-side of the fetus, rinsed in ice-cold DEPC-treated water and cut into small pieces. Every skin sample was divided into two parts; one was stained and one was immediately frozen in liquid nitrogen for RNA-seq and qPCR analyses. All experimental procedures and the study design were carried out in accordance with the Care and Use of Laboratory Animals (Ministry of Science and Technology of China, 2006), and were approved by the Experimental Animal Manage Committee of Northwest A&F University under contract (NWAFU-31402038).

### Skin staining

Skin tissues were prepared for histological sectioning, using the procedures Carter and Clarke (1957) [21] described. Skin samples from the different fetuses were placed in centrifuge tubes containing 4% paraformaldehyde solution (made with 0.1 M sodium phosphate buffer, pH = 7.4). Samples were then placed in small individual baskets and dehydrated through a series of graded ethanol. Processed skin samples were then embedded in paraffin, and serial vertical sections of skin were cut at a thickness of 5 μm. Sections were stained using the special tetrachrome stain ‘sacpic’ [22]. The sacpic method was previously used to determine the activity state of HF during postnatal periods [23].

### Total RNA isolation, library construction and sequencing

Total RNA was isolated, using Trizol Reagent (Invitrogen) according to the manufacturer’s instructions, from each skin sample after grinding the sample in liquid nitrogen. The quality and concentration of the total RNA were determined using an Agilent 2100 Bioanalyzer (Agilent). RNA samples were stored at -80°C for later library construction and sequencing.

Nine RNA libraries for each skin sample were constructed, representing samples from the three time points during HF development. The libraries were as follows: E60_1, E60_2 and E60_3 as replicate libraries for the E60 experimental group, E120_1, E120_2 and E120_3 for the E120 experimental group, and NB_1, NB_2 and NB_3 for the NB experimental group. Oligo (dTs) were used to isolate poly (A) mRNA. The mRNA was fragmented and reverse transcribed using random primers. Second-strand cDNAs were synthesised using RNase H and DNA polymerase I. The double-strand cDNAs were then purified using the QiaQuick PCR extraction kit. The required fragments were purified via agarose gel electrophoresis and were enriched through PCR amplification. Finally, the amplified fragments were sequenced using Illumina HiSeq^™^ 2000 (GeneDenovo Co., Guangzhou, China) according to the manufacturer’s specifications. The raw sequencing data were submitted to the NCBI SRA database under access number SRP059481.

### Mapping reads to the reference genome

The original sequencing-received image data were transferred into sequence data via base calling, which is defined as raw data or raw reads stored in the fastq format. Raw reads of all nine samples were pre-processed through the removal of containing adaptors-reads with more than 5% unknown nucleotides. Low-quality reads were also removed, in which the percentage of low-quality bases of quality value ≤ 5 was more than 50% in a read. The clean reads of each stage were aligned to the goat genome assembly CHIR_1.0 [24] using SOAP aligner/soap2. Mismatches of no more than two bases were allowed in the alignment, and uniquely mapped reads were obtained.

### Expression annotation

For gene expression analysis, the number of unique-match reads was calculated and normalised to RPKM (reads per kb per million reads). Expression levels of each gene between two groups were compared to give an expression difference using DESeq, as Abders and Huber [25] described. The *P* value corresponded to differential gene expression at statistically significant levels [26]. FDR (False Discovery Rate) was used to determine the *P* value threshold. DEGs were defined as FDR ≤ 0.05 and absolute value of log_2_^Ratio^ ≥ 1.

Gene expression data were normalised to 0, log_2_^(E120/E60)^ and log_2_^(NB/E60)^. The union set of all DEGs was used for further expression pattern analysis. STEM (Short Time-series Expression Miner, v1.3.8) was used to profile the gene set into eight expression patterns. Cluster profiles with a *q* value ≤ 0.05 were considered significantly expressed. GO (gene ontology) annotation was analysed using Blast2GO software (version 2.3.5) (https://www.blast2go.com/). Functional classification of the DEGs was performed using WEGO software. The KEGG (kyoto encyclopaedia of genes and genomes) pathway annotation was carried out using Blastall software against the KEGG database (http://www.kegg.jp/).

### Confirmation of RNA-seq results with qPCR

First-strand cDNA was generated from 1 μg total RNA isolated from the skin samples using the Superscript^™^ First-strand Synthesis System (Invitrogen). To confirm the transcriptomic analysis results, eight genes were chosen consistent with those derived from sequencing, and were subjected to qPCR (quantitative reverse transcription PCR), including *DNER* (delta notch-like EGF repeat containing), *PRSS35* (protease serine 35), *HOXC13* (homebox C13), *TRPV3* (transient receptor potential cation channel subfamily V member 3), *BREVICAN*, *MYOG* (myogenin), cystatin-M, and cytochrome P450 4X1-like. *ACTB* (beta-actin) was chosen as an internal reference gene since it had an equal RPKM value among the three stages. The qPCR was carried out on a CFX Connect Real-time PCR Detection System (Bio-Rad) using SYBR Premix Ex Taq (TaKaRa). The qPCR was run as follows: 50°C for 2 min and 95°C for 10 min, followed by 45 cycles of 95°C for 15 s, 60°C for 1 min, and 72°C for 45 s. Each qPCR analysis was performed in triplicate. Relative gene expression levels were calculated using the 2^-△△Ct^ method [27]. The primers used for qPCR are listed in S1 Table.

### Immunohistochemistry

Immunohistochemical staining was performed using a rabbit super-sensitive two-step immunohistochemical detection kit (PV-9001; Zhongshan Goldenbridge Biochemistry, Co., Ltd., Beijing, China). According to the manufacturer’s instructions, paraffin-embedded blocks were sectioned at 5 μm, and then were deparaffinized and rehydrated. After a boiling pre-treatment in the citrate buffer (pH 6.0) for antigen retrieval, slides were immersed in 3% hydrogen peroxide for 10 min to remove the endogenous peroxidase activity, blocked with blocking reagent in normal goat serum (ZLI-9020, ZSGB-BIO, Beijing, China) for 2 hours. They were then incubated at 4°C overnight in a humidified chamber with the following primary antibodies: VDR (vitamin D receptor) (rabbit, 1:300; Boster, Wuhan, Hubei, China), KLK11 (kallikrein 11) (rabbit, 1:100; Boster, Wuhan, Hubei, China). After being washed three times with PBS for 5 min each time, the samples were treated with a secondary antibody (PV-9002; Zhongshan Goldenbridge Biotechnology Co., Ltd., Beijing, China) at 37°C for 20 min. Then, the samples were washed three times with PBS for 5 min each time, and were treated with DBA for 1 min, counterstained with haematoxylin and countered under light microscopy.

## Results

### Establishment of the stages of HF morphogenesis

**Fig 1:**
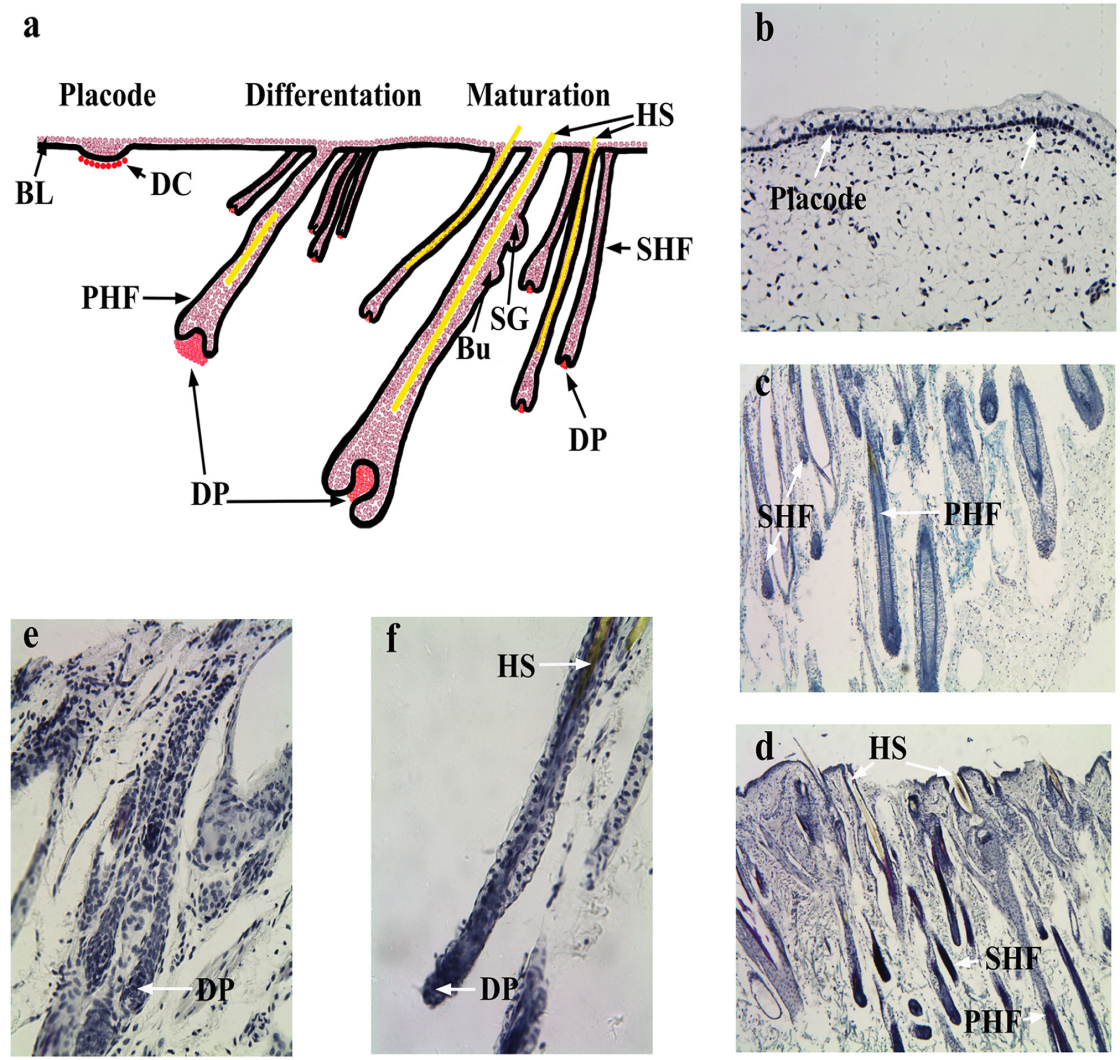
SACPIC staining of fetal goat tissue. (a) Schematic representation of the process of HF morphogenesis. Longitudinal sections (× 40) of fetal HF at (b) placode (E60), (c) differentiation stage (E120), and (d) maturation stage (NB). A high magnification view (× 160) of HF at (e) E120 and (f) NB. BL: Basal layer, DC: Dermal condensate, PHF: Primary hair follicle, SHF: Secondary hair follicle, DP: Dermal papilla, Bu: Bugle, SG: Sebaceous gland, HS: Hair shaft.

The time point and characteristics of HF morphogenesis during embryogenesis in cashmere goats has been reported to be between 55 and 135 days of fetal life, and this process is divided into three specific stages: initiation, differentiation and maturation [7]. Our histological study demonstrated that mesenchymal cells began to accumulate at E60 and the basal cells formed a visible hair germ (Fig 1a and 1b). At E120, PHF down-growth reached the subcutis and formed hair shafts at the upper end. A large number of SHF branched out around the PHF and a hair road was formed. A large number of SHF underwent rapid cytodifferentiation (Fig 1a, 1c and 1e). At NB, the PHF acquired their maximal length and prominent hair/cashmere shafts emerged through the epidermis. The PHF and a small amount of SHF were then matured (Fig 1a, 1d and 1f). In addition, there are no clear morphological structure differences of HF at E120 and NB. Our results are consistent with previous findings showing that the development of SHF demonstrated a hysteresis effect [7]. The process of HF development was found to have overlapping morphological states: HF initiation, differentiation of PHF and SHF, and the maturation of PHF.

### Identification of expressed transcripts in the skin transcriptome

To quantify the gene expression patterns of fetal skin samples, we constructed nine cDNA libraries and then subjected them to deep sequencing using Illumina HiSeq2000. From each library, we obtained over 21.1 million raw reads, which was sufficient for a quantitative analysis of the gene expression patterns. After filtering the adaptor sequences, regions containing N sequences and low quality sequences, over 20.6 million clean reads were generated in each library. The percentage of clean reads among raw tags in each library ranged from 97.52% to 97.86% (S1 Fig), indicating that high quality RNA-seq data were obtained for further analysis. The clean transcripts obtained were then used for further analysis. An average of 15.3 million reads per sample was mapped to the goat genome (range: 12.8–19.5 million). Of the total reads, the rate of match reads was more than 62%, and the remaining reads were unmatched (S2 Table).

Gene coverage is the percentage of a gene covered by reads. The similarity distribution showed a comparable pattern with approximately 40% of sequences having a similarity of 80% from three biological replicates, suggesting good reproducibility of this method (S2 Fig). A correlation analysis of two parallel samples provided an evaluation of the reliability of experimental results and rationality of sampling. In our study, the scatter plot showed that the correlation values of two biological replicates at each stage were up to 0.92 (R^2^ ≥ 0.92) based on the RPKM values, suggesting sufficient reproducibility and rationality of sampling (S3 Fig).

### Differentially expressed genes at E60, E120 and NB

To better examine the biological mechanism of HF morphogenesis, it is important to identify the DEGs at each stage. Mean RPKM values were generated out of three biological replicates of each experimental group. Expression levels of a distinct gene from two groups were compared to give an expression difference using the DESeq [25]. The number of DEGs in E60 vs. E120, E120 vs. NB, and E60 vs. NB was 1,024, 0 and 1,801, respectively, for transcripts detected with |log_2_^Ratio^| ≥ 1.0 and *q* value ≤ 0.05. Furthermore, no DEGs in E120 vs. NB were identified, suggesting that the E120 and NB have similar transcript profiles. A total of 2,059 DEGs were found in E60 vs. E120 and E60 vs. NB (S3 Table).

Our analysis identified a set of genes belonging to keratin family member encoding genes (*KRT*) and keratin-associated protein encoding genes (*KRTAP*), which were markedly up-regulated in E120 vs. E60. Studies have shown that *KRT* and *KRTAP* are major structural proteins of the hair fibre and sheath, and their content is important for fleece quality [28, 29]. In addition, we compared our results with RNA transcriptomic data from 20–50 SHF or PHF in cashmere goats. Of these, 49 *KRT* genes and 30 *KRTAP* genes were annotated in the goat genome [24]. We found that more than half of *KRT* and *KRTAP* (21/31) were detectable in the HF of cashmere goats (Table 1). Most of the *KRT* and *KRTAP* genes were evolutionarily conserved, however the expression patterns in HF present some differences among different species, such as between humans and sheep, due to the distinctive features of hair and wool [30]. Thus, our results could be useful in finding the expression patterns of *KRT* and *KRTAP* in the HF of cashmere goats, whose composition and interactions could determine the fibre properties.

**Table 1:** List of *KRT* and *KRTAP* genes differentially expressed in E120 vs. E60 and NB vs. E60, which were also detectable in the HF of cashmere goats.

Gene_Symbol	RPKM-E60	RPKM-E120	RPKM-NB	Gene_description
KRTAP3-1	4.427	7439.151	9764.394	keratin associated protein 3–1
LOC102182256	0.426	1613.334	920.042	keratin-associated protein 26-1-like
KRTAP11-1	2.304	3621.071	4096.327	keratin associated protein 11–1
LOC102170546	0.862	2019.933	2212.462	keratin-associated protein 3-3-like
LOC102179881	1.222	1352.297	2038.735	Keratin type I microfibrillar 47.6 kDa-like
LOC102184223	0.398	839.849	793.224	Keratin type II microfibrillar component 5-like transcript variant X1
KRT28	0.290	132.397	146.953	keratin 28
KRT25	3.660	2884.336	3897.065	keratin 25
KRT14	0.704	264.622	325.542	keratin 14 transcript variant X1
LOC100861181	1.598	1121.897	2878.802	keratin associated protein 7.1
LOC100861175	0.966	2365.912	2423.162	keratin associated protein 13.1
LOC102185436	3.410	6408.016	6051.503	Keratin type II microfibrillar component 7C-like
KRT27	2.347	2187.778	3154.963	keratin 27
LOC100860930	0.636	553.624	1562.745	high-glycine tyrosine keratin type II.6
LOC102183211	1.512	2797.552	2841.306	Keratin type II cuticular Hb1-like
LOC102178766	50.833	116.058	224.967	Keratin type I cytoskeletal 19-like transcript variant X2
KRT80	3.702	152.787	146.397	keratin 80
KRT84	0.331	149.689	77.136	keratin 84
KRT82	0.095	123.955	70.889	keratin 82 transcript variant X1
LOC102176997	4.612	223.815	330.079	keratin type I cytoskeletal 42-like
KRT23	0.165	116.912	117.280	keratin 23 histone deacetylase inducible

Moreover, the following genes were up-regulated: *LRRC15* (leucine-rich repeat-containing protein 15), which may function in cell communication; *LHX2* (LIM homeobox 2), which maintains stem cell characteristics in HF; *VSIG8* (V-set and immunoglobulin domain-containing protein 8), which is enriched in the cuticle; and *PRR9* (proline-rich protein 9), which serves as substrates for transglutaminases that are responsible for cross-linking (S3 Table) [31, 32]. This indicated that these DEGs are associated with structural protein biosynthesis during HF and skin development.

### Validation of the sequencing data by quantitative PCR

**Fig 2:**
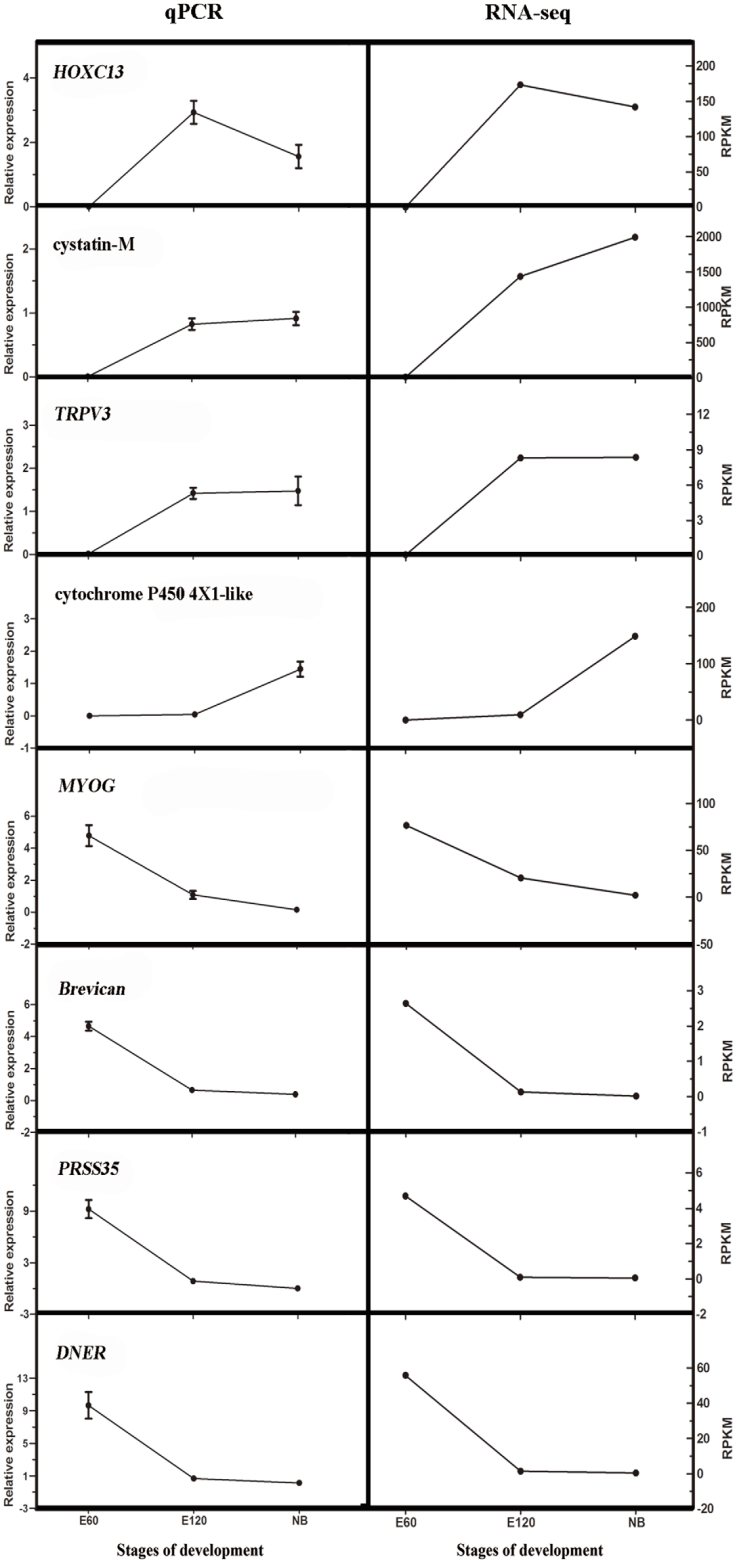
Expression levels of tested reference genes revealed by qPCR and RNA-seq. Data from qPCR are shown as means ± standard error (SE) of three replicates. RPKM from RNA-seq are shown as means and SE of three replicates. The left side indicates the data from qPCR; the right side shows RPKM from RNA-seq.

To validate the accuracy and reproducibility of the transcriptome results, we selected eight genes for qPCR validation (Fig 2). Linear regression analysis of these gene expression ratios between RNA-seq and qPCR was highly correlated (r^2^ = 0.90) (S4 Fig). Hence, these qPCR results confirmed that our RNA-seq findings provided reliable data.

### Immunostaining of KLK11 and VDR

**Fig 3:**
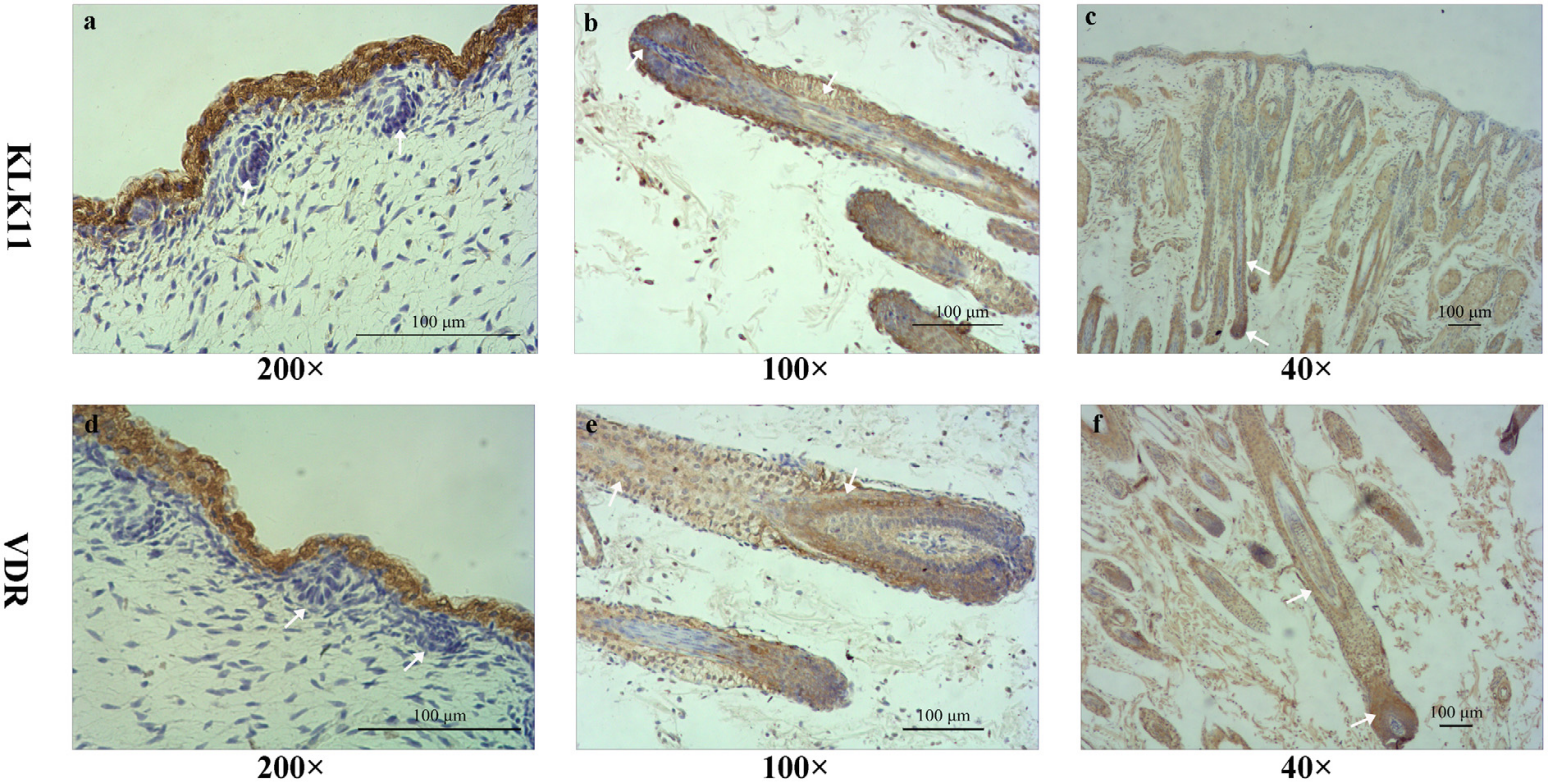
Distribution of KLK11 and VDR immunoreactivity in fetal goat skin. (a) E60: KLK11 was detected in the epidermis. The placode of HF shows little or no immunoreactivity at this stage (arrow). (b) E120: KLK11 was expressed strongly in the root sheaths and slightly in the bulb of HF (arrow). (c) NB: KLK11 was present in the hair matrix cells and root sheaths (arrow). (d) E60: VDR shows intense immunostaining in the epidermis. Very little VDR immunoreactivity was detected in the placode (arrow). (e) E120: VDR immunoreactivity is predominantly detected in the ORS keratinocytes and the bulb of HF (arrow). (f) NB: In fully formed HF, VDR staining was present in the root sheaths and bulb cells, where immunoreactivity was particularly enhanced in the ORS keratinocyte and matrix zone. Bar: 100 μm.

The distribution of KLK11 and VDR was not detected in the placode at E60 (Fig 3a and 3d). The immunoreactivity increased as follicle development progressed. KLK11 immunoreactivity was localised to the nucleus of cells of the root sheaths and bulb of HF at E120 and NB (Fig 3b and 3c). VDR immunoreactivity was particularly enhanced in the ORS keratinocyte and matrix zone at E120 and NB (Fig 3e and 3f).

### GO-term analysis of DEGs

**Fig 4:**
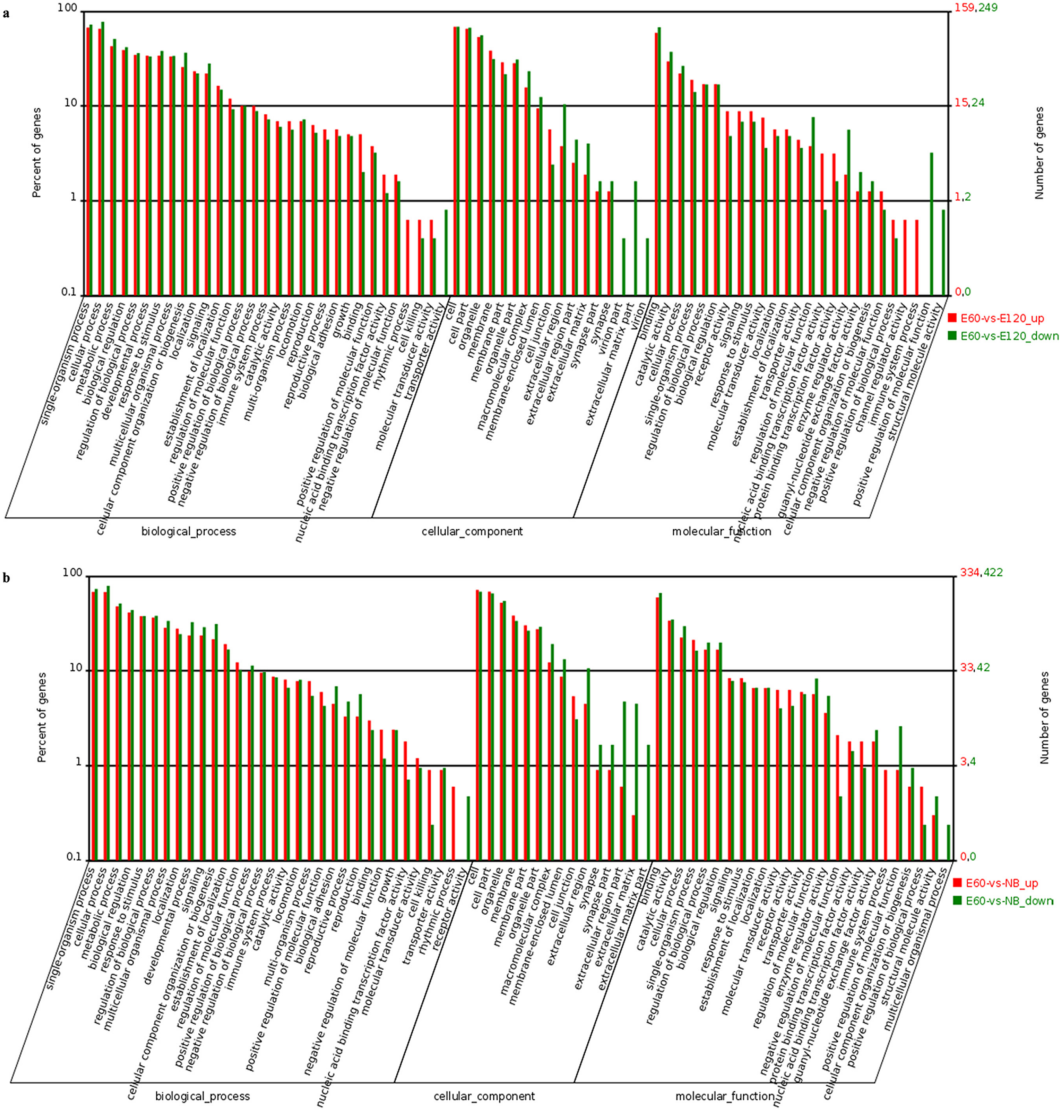
Functional categorisation of differentially expressed genes among libraries. The results are summarised in three main categories: biological process, cellular component and molecular function. The X-axis indicates the second level term of gene ontology; The Y-axis shows the percentage of genes.

To better understand the biological behaviour of HF and skin morphogenesis, 2,059 DEGs were categorised into three gene ontology categories: cellular component, biological process, and molecular function. DEGs between E60 vs. NB and E60 vs. E120 were categorised into 72 and 73 functional groups based on sequence homology. The top five functional categories of up-regulation DEGs in E60 vs. E120 included cell, single-organism process, cellular process, cell part and binding. The top five functional categories of up-regulation DEGs in E60 vs. NB included cell, cell part, single-organism process, cellular process and binding (Fig 4).

Under the cellular component category, a large number of up-regulation DEGs, as well as down-regulation DEGs, was categorised as cell part, cell and organelle in E60 vs. NB and in E60 vs. E120 (Fig 4). The cellular and developmental process in the biological process, binding and catalytic activity in molecular function were found to be greater in E60 vs. NB than in E60 vs. E120 (Fig 4). Further enrichment analysis was found to be related to the development process and signal transduction, and the detected DEGs were enriched in different terms related to HF and skin development. For example, the DEGs *LHX2*, *BMP4* (bone morphogenetic protein 4), *S100A1* (S100 calcium binding protein A1), *ECM1* (extracellular matrix protein 1) and *WIF1* (WNT inhibitory factor 1) were enriched in the transcriptional activator, secreted term, binding protein, glycoprotein and secreted protein, respectively.

### KEGG pathway enrichment analysis of DEGs

KEGG analysis predicted that 55.5% (1,142/2,059) of DEGs were involved in 218 pathways. The top 20 KEGG pathways with the highest representation of DEGs are listed in Table 2. The maps with the highest DEGs representation were metabolic pathways (141 DEGs, 12.35%). “Pathways in cancer” (64 DEGs, 5.60%) was the first significantly highly enriched (*q* value ≤ 0.05). “Pathways in cancer” is a collection of many pathways and has an important functions in promoting cell proliferation and evading apoptosis. The cell adhesion molecules, axon guidance, pathogenic Escherichia coli infection, tight junction, phagosome, leukocyte transendothelial migration and ECM (extra-cellular matrix)-receptor interaction are also significantly enriched, respectively (S4 Table). From these pathways, information on communication between and within the epidermis and dermis, which regulates HF development, can be obtained. As an example, there are two pathways for epidermis and dermis communication: cell adhesion molecules and ECM-receptor interaction pathway. DEGs in the cell adhesion molecules pathway is to mould, relax or reinforce cell contacts in areas of increased HF morphogenetic activity, such as *NCAM1* (neural cell adhesion molecule 1) and *CDH1* (cadherin 1, type 1, E-cadherin epithelial) [33]. ECM-receptor interaction pathways are important to HF morphogenesis because they affect cell physiological activities, including proliferation and migration [34, 35]. These annotations provided a good platform for further research into understanding EMI in the HF and skin development process.

**Table 2:** 20 top KEGG pathways with high representation of the DEGs in E120 vs. E60 and NB vs. E60.

Pathways_level3	All_profiles (1142)	profile0 (213)	profile1 (318)	profile6 (482)	profile7 (89)
Metabolic pathways	141(12.35%)	23(10.80%)	26(8.18%)	68(14.11%)	20(22.47%)
Pathways in cancer	64(5.60%)	16(7.51%)	18(5.66%)	23(4.77%)	5(5.62%)
Focal adhesion	37(3.24%)	9(4.23%)	14(4.40%)	11(2.28%)	1(1.12%)
Regulation of actin cytoskeleton	37(3.24%)	5(2.35%)	13(4.09%)	17(3.53%)	2(2.25%)
Cell adhesion molecules (CAMs)	35(3.06%)	8(3.76%)	10(3.14%)	11(2.28%)	5(5.62%)
Axon guidance	34(2.98%)	8(3.76%)	11(3.46%)	14(2.90%)	1(1.12%)
Endocytosis	33(2.89%)	7(3.29%)	9(2.83%)	14(2.90%)	0(0.00%)
MAPK signaling pathway	33(2.89%)	10(4.69%)	6(1.89%)	15(3.11%)	2(2.25%)
Pathogenic Escherichia coli infection	32(2.80%)	1(0.47%)	5(1.57%)	21(4.36%)	5(5.62%)
Calcium signaling pathway	30(2.63%)	5(2.35%)	5(1.57%)	14(2.90%)	4(4.49%)
Tight junction	30(2.63%)	1(0.47%)	7(2.20%)	20(4.15%)	2(2.25%)
Chemokine signaling pathway	29(2.54%)	7(3.29%)	7(2.20%)	12(2.49%)	0(0.00%)
Phagosome	28(2.45%)	3(1.41%)	9(2.83%)	10(2.07%)	6(6.74%)
Leukocyte transendothelial migration	26(2.28%)	1(0.47%)	5(1.57%)	16(3.32%)	3(3.37%)
Neuroactive ligand-receptor interaction	25(2.19%)	1(0.47%)	7(2.20%)	11(2.28%)	4(4.49%)
Glutamatergic synapse	24(2.10%)	8(3.76%)	3(0.94%)	9(1.87%)	3(3.37%)
ECM-receptor interaction	23(2.01%)	7(3.29%)	9(2.83%)	5(1.04%)	1(1.12%)
Cytokine-cytokine receptor interaction	23(2.01%)	5(2.35%)	5(1.57%)	8(1.66%)	2(2.25%)
Purine metabolism	23(2.01%)	3(1.41%)	12(3.77%)	6(1.24%)	1(1.12%)
Amoebiasis	23(2.01%)	6(2.82%)	5(1.57%)	7(1.45%)	4(4.49%)

### Expression profiling among the three developmental stages

To determine the primary gene expression trajectories, we further conducted a clustering analysis of all 2,059 DEGs. These genes could be clustered into eight profiles based on their expression modulation via STEM software, in which 1,975 were clustered into four profiles (*P* value ≤ 0.05), including two down-regulated patterns (profile1 and profile0) and two up-regulated patterns (profile6 and profile7) (S5 Fig). Profile6 and profile1 contained genes modulated after E120, and profile0 and profile7 contained genes positively or negatively modulated along the whole time course (S5 Fig). Profile1 and profile0 contained 574 and 449 DEGs, respectively, while profile6 and profile7 contained 812 and 140 DEGs (S5 Fig). The results offer new information related to further characterization of novel molecules associated with HF and skin development in cashmere goats, and their expression profiles were characterised in detail.

**Fig 5:**
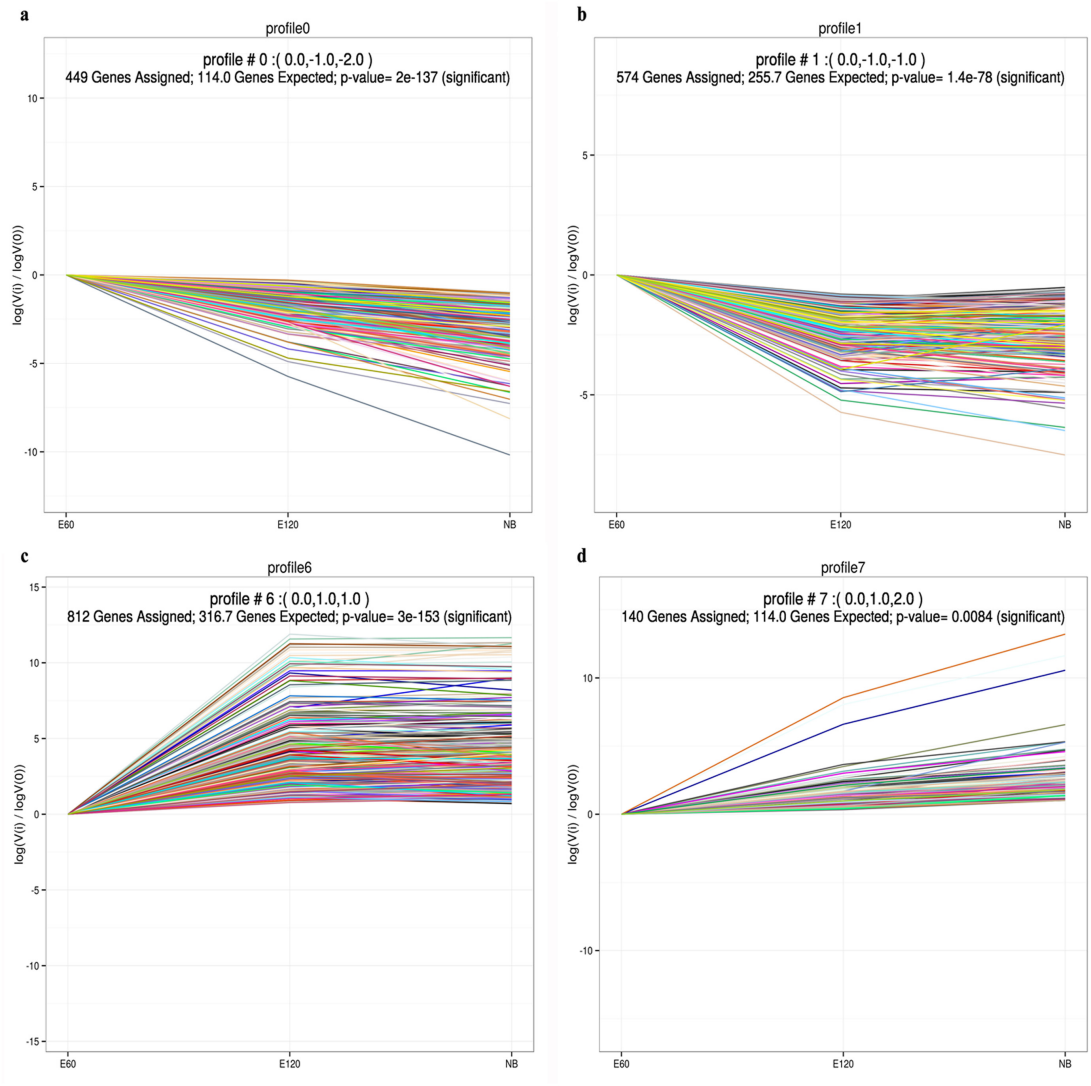
Cluster trajectory profiles across stages of HF development. Profiles a-d: Each X-axis indicates the HF development state (E60, E120, and NB); The Y-axis shows expression changes. Trajectory cluster analyses (see Methods) show that gene expression did not steadily increase or decrease in the progression. Four profiles (profiles a-d) represent the major transcriptional trajectories.

The most abundant group was profile6, with 1,203 genes up-regulated in E120 samples, compared to E60 samples, before reaching steady-state (Fig 5c). At E120, a number of new SHF grew rapidly as branches of mature PHF. These up-regulated genes may be associated with SHF cytodifferentiation and PHF maturation. *KRT* and *KRTAP* are expressed in the HF of sheep and humans, which are abundantly expressed during HF differentiation [28, 30, 36, 37]. A total of 30 up-regulated genes belonged to the *KRT* and *KRTAP*, such as *KRT34*, *KRT33a*, and *KRT33b* (S5 Table). These data were in agreement, showing that hair keratinocyte proliferation was active after E120, and these genes may be essential in determining the structure and quality of cashmere fibres. Amino acid metabolism was significantly important to protein synthesis. Sulphur-containing amino acids stimulate proliferation of hair forming cells in the HF, including methionine, cysteine and cystine [38]. In the cysteine and methionine metabolism, four genes were annotated to profile6, including *AHCY* (adenosylhomocysteinase) (S5 Table). AHCY is a ubiquitous enzyme that catalyses the hydrolysis of S-adenosylhomocysteine to adenosine and homocysteine [39]. Adenosine and homocysteine indicate an important branch point in the metabolism of methionine and cysteine, and adenosine could stimulate the expression of *FGF7* (fibroblast growth factor 7) in dermal papilla (DP) cells [40]. Methionine is a nutritionally essential amino acid for cashmere fibre production, and cysteine is also required to produce maximum growth [38]. These DEGs in cysteine and methionine metabolism contributed to HF and skin protein metabolism in cashmere goats.

We also found that genes associated with the immune system and infectious diseases did not reach peak expression until HF morphogenesis was almost completed in profile7 (Fig 5d). Evidence in mice and humans indicates that the HF in the anagen phase is an immune-privileged organ, which expressed at very low levels of Class II MHC class antigens [41] (S5 Table). Expression of these genes provides strong evidence that immune privilege is crucial during PHF initiation.

In the metabolic pathways, six DEGs were clustered to profile0 or profile1 showing down-regulated trends. They encode enzymes that regulate GAG (glycosaminoglycan) biosynthesis-keratin sulfate, including *B4GALT4* (UDP-Gal:betaGlcNAc beta 1,4-galactosyltransferase, polypeptide 4), *B4GALT2*, and *B3GNT1* (UDP-GlcNAc:betaGal beta-1,3-N-acetylglucosaminyltransferase 1) (S5 Table). In rodent models, GAG distribution around the hair matrix is essential for hair morphogenesis [42, 43]. Keratin sulfate is expressed in the keratinocytes of epithelial tissue and the connective tissue sheath of adult HF [44]. All of the aforementioned evidence suggests an important role for keratin sulfate activation at the time of placode formation.

In the ECM-receptor interaction pathway, 16 out of 23 DEGs were clustered to profile0 or profile1, showing down-regulated trends (Fig 5a and 5b). ECM is composed of fibrous structural proteins, for example, collagens and laminin, and matricellular proteins, for example, thrombospondin and tenascins, which modulate EMI and HF morphogenesis [34, 45]. Many of these ECM genes were observed in our DEG datasets, including *COL5A2* (collagen type V, alpha 2), *THBS4* (thrombospondin 4) and *TNC* (tenascin C) (S5 Table). We assume that the enhanced expression of genes plays multiple roles in PHF initiation.

## Discussion

The yield and diameter of fleece in certain mammal species are thought to be determined by the number and density of HF, which are established in the early stages of fetal life [3]. However, HF development is a complex process, and the processes and developmental mechanisms of fleece production at different stages in cashmere goats remain largely uncharacterised. Previous studies have confirmed that three specific developmental stages are typical (E60, E120 and NB) during the time interval spanning from initiation to maturation of HF using sacpic staining [7]. Therefore RNA-seq was conducted using skin tissues from both fetal and newborn goats. Fibre diameter is highly correlated with the size of the dermal papilla cells, whose origin can be traced to the earliest features of the developing HF.

HF initiation is thought to be partially influenced by interactions of ECM-receptor components [34]. ECM-receptors consist of integrins, proteoglycans and other transmembrane molecules that mediate the ECM interactions with cells [45]. Integrins have been shown to be expressed in mesenchymal aggregates of developing HF [46]. We found that three α-integrin genes were highly expressed at E60: α5, α9 and α11 (Table 3, S6 Fig). Integrins frequently act synergistically with other growth factor receptors [47, 48]. We also discovered that two corresponding growth factor receptors genes were highly expressed at E60, including *FRS3* (fibroblast growth factor receptor substrate 3) and *FGFR1* (fibroblast growth factor receptor 1) (Table 3, S6 Fig). These findings were in line with the DP and skin high-throughput transcriptome sequencing results obtained during mature HF cycling phases, indicating that these genes probably have a role in HF EMI, and they might be the mediators of HF initiation in cashmere goats [19, 49].

**Table 3:** Selected genes differentially expressed in cashmere goat skin during the HF initiation.

Class	Gene	Gene Name	log_2_ Fold Change		*P* value		FDR	
			E120/E60	NB/E60	E120/E60	NB/E60	E120/E60	NB/E60
ECM-receptor interaction								
	*ITGA5*	integrin alpha 5	-1.200	-1.280	0.0006	0.0004	0.0247	0.0134
	*ITGA11*	integrin alpha-11	-	-1.653	-	0.0057	-	0.0417
	*ITGA9*	Integrin, alpha-9	-2.152	-2.799	0.0007	0.0003	0.0258	0.0124
	*THBS3*	thrombospondin 3	-1.785	-1.807	0.0033	0.0027	0.0438	0.0293
	*THBS2*	thrombospondin 2	-	-1.261	-	0.0016	-	0.0233
	*THBS4*	thrombospondin-4	-	-2.820	-	0.0071	-	0.0470
	*SDC3*	syndecan 3	-1.123	-2.062	0.0006	0.0001	0.0246	0.0088
	*COL5A3*	collagen type V alpha 3	-1.781	-1.859	0.0034	0.0032	0.0441	0.0318
	*COL5A2*	collagen type V alpha 2	-1.975	-2.354	0.0028	0.0012	0.0415	0.0208
	*COL5A1*	collagen type V alpha 1	-2.294	-2.714	0.0001	0.0000	0.0147	0.0037
	*HSPG2*	heparan sulfate proteoglycan 2	-	-1.334	-	0.0035	-	0.0330
	*TNC*	tenascin	-4.707	-4.900	0.0003	0.0003	0.0198	0.0114
Glycosaminoglycan biosynthesis—keratan sulfate								
	*B4GALT4*	UDP-Gal:betaGlcNAc beta 1,4- galactosyltransferase, polypeptide 4, transcript variant X1	-1.500	-1.910	0.0007	0.0001	0.0262	0.0088
	*ST3GAL1*	ST3 beta-galactoside alpha-2,3-sialyltransferase 1, transcript variant X1	-1.740	-2.160	0.0038	0.0048	0.0460	0.0385
	*ST3GAL1*	ST3 beta-galactoside alpha-2,3-sialyltransferase 1, transcript variant X2	-	-3.030	-	0.0046	-	0.0375
growth factor receptors								
	*FRS3*	fibroblast growth factor receptor substrate 3	-	-1.211	-	0.0035	-	0.0331
	*FGFR1*	fibroblast growth factor receptor 1 isoform X6	-	-1.218	-	0.0002	-	0.0114

Some transcriptional regulator genes for HF protein synthesis were also remarkably up-regulated at E120 and NB, such as *GPRC5D* (G-protein coupled receptor family C group 5 member D) [50], *PADI3* (protein-arginine deiminase type-3) [51], *HOXC13* [52], *FOXN1* (forkhead box protein N1) [53], and *MSX*2 (msh homeobox 2) [54] (Table 4, S6 Fig). This suggests that these genes take part in the keratinization process accompanied by basal regulation of the inner root sheath and hair shaft formation in cashmere goats. Here, we observed that Wnt-related genes were up-regulated at E120 and NB, including *WNT10A* (wingless-type MMTV integration site family, member 10A), *WNT11*, and β-catenin (Table 4, S6 Fig), implying that these genes probably play critical roles in HF cytodifferentiation and maturation. In addition, one other major signalling transduction pathway, TGF-beta (transforming growth factor-b)/BMP (bone morphogenetic protein), has recently been shown to play a central role in HF and skin development [14]. The Bmp4/Bmp2/Msx2/Foxn1 acidic hair keratin pathway is involved in the control of hair shaft growth and differentiation, and TGF-beta/BMP signals are necessary for regulating hair shaft growth and differentiation [5, 54, 55].

**Table 4:** Selected genes differentially expressed in cashmere goat skin during the HF cytodifferentiation and maturation.

Class	Gene	Gene Name	log_2_ Fold Change		*P* value		FDR	
			E120/E60	NB/E60	E120/E60	NB/E60	E120/E60	NB/E60
Hair follicle and transcriptional regulator genes								
	*CST6*	cystatin E/M, mRNA	7.24	7.71	0.0003	0.0029	0.0212	0.0304
	*DSG4*	desmoglein 4	10.48	10.53	0.0000	0.0027	0.0109	0.0294
	*FOXN1*	forkhead box N1	-	7.10	-	0.0037	-	0.0341
	*LHX2*	LIM homeobox 2	7.82	7.53	0.0012	0.0006	0.0310	0.0159
	*HOXC13*	homebox C13	9.68	9.39	0.0036	0.0007	0.0451	0.0170
	*PRR9*	proline rich 9	9.66	10.02	0.0003	0.0008	0.0202	0.0175
	*VSIG8*	V-set and immunoglobulin domain-containing 8	-	8.20	-	0.0004	-	0.0137
	*PADI3*	peptidylarginine deiminase, type III	9.94	9.75	0.0043	0.0011	0.0484	0.0199
Wnt related								
	*SFRP5*	secreted frizzled-related protein 5	4.35	4.57	0.0000	0.0023	0.0112	0.0273
	*CHP2*	calcineurin-like EF-hand protein 2	1.53	-	0.0045	-	0.0494	-
	*CTNNB1*	catenin cadherin-associated protein beta 1 transcript variant X3	-	1.11	-	0.0044	-	0.0367
	*FZD10*	frizzled family receptor 10	-	1.58	-	0.0042	-	0.0362
	*CTNNBIP1*	catenin, beta interacting protein 1, transcript variant X1	2.26	2.46	0.0006	0.0055	0.0242	0.0413
	*WNT11*	wingless-type MMTV integration site family, member 11	-	1.31	-	0.0010	-	0.0193
	*TCF7*	transcription factor 7 (T-cell specific, HMG-box), transcript variant X4	1.46	-	0.0000	-	0.0045	-
	*WIF1*	WNT inhibitory factor 1, transcript variant X1	5.13	-	0.0023	-	0.0385	-
	*WNT10A*	wingless-type MMTV integration site family, member 10A	-	1.79	-	0.0029	-	0.0301
	*FZD5*	frizzled family receptor 5	3.20	-	0.0016	-	0.0335	-
TGF-beta/BMP								
	*SMAD6*	SMAD family member 6	1.40	1.05	0.0008	0.0014	0.0268	0.0221
	*SMAD7*	SMAD family member 7 transcript variant X2	1.70	-	0.0030	-	0.0424	-
	*PPP2R1B*	protein phosphatase 2 regulatory subunit A beta	2.64	2.24	0.0020	0.0032	0.0373	0.0319
	*BMP2*	bone morphogenetic protein 2	2.87	3.53	0.0016	0.0034	0.0335	0.0329
	*ID2*	inhibitor of DNA binding 2 dominant negative helix-loop-helix protein	1.57	1.28	0.0016	0.0008	0.0336	0.0175
	*LTBP2*	latent transforming growth factor beta binding protein 2	-	1.13	-	0.0054	-	0.0409
	*BMP4*	bone morphogenetic protein 4 transcript variant X1	2.32	1.84	0.0010	0.0004	0.0293	0.0126
	*CDKN2B*	cyclin-dependent kinase inhibitor 2B	3.83	3.49	0.0022	0.0002	0.0383	0.0112
	*LOC102187369*	bone morphogenetic protein 8A-like	5.16	4.98	0.0035	0.0032	0.0442	0.0320
	*INHBB*	inhibin beta B	-	1.73	-	0.0066	-	0.0454
Notch related								
	*DTX2*	deltex homolog 2	1.29	1.50	0.0027	0.0012	0.0409	0.0205
	*JAG2*	jagged 2	1.19	1.00	0.0023	0.0065	0.0391	0.0450
	*JAG1*	jagged-1	1.53	1.21	0.0024	0.0024	0.0397	0.0277

Expression of TGF-beta/BMP-related genes was also increased during the late stages of HF and skin morphogenesis, including *BMP2*, *BMP4* and *BMP8A*. We also found that some BMP inhibitors were up-regulated in E120 skin, such as *SMAD6* and *SMAD7*, which are important for antagonising TGF-beta/BMP activity and balance BMP inhibition [56] (Table 4, S6 Fig). Notch signalling controls DP signature molecules, which in return signal to the matrix cells to promote HF differentiation [5]. We noticed that up-regulated genes were related to Notch signalling, including *JAG1* (jagged-1) (Table 4, S6 Fig). *JAG1* is expressed in pre-cortex cells and the cuticle layer of the inner root sheath [57]. We believe that these annotated genes are involved in HF cytodifferentiation and maturation in cashmere goats.

**Fig 6:**
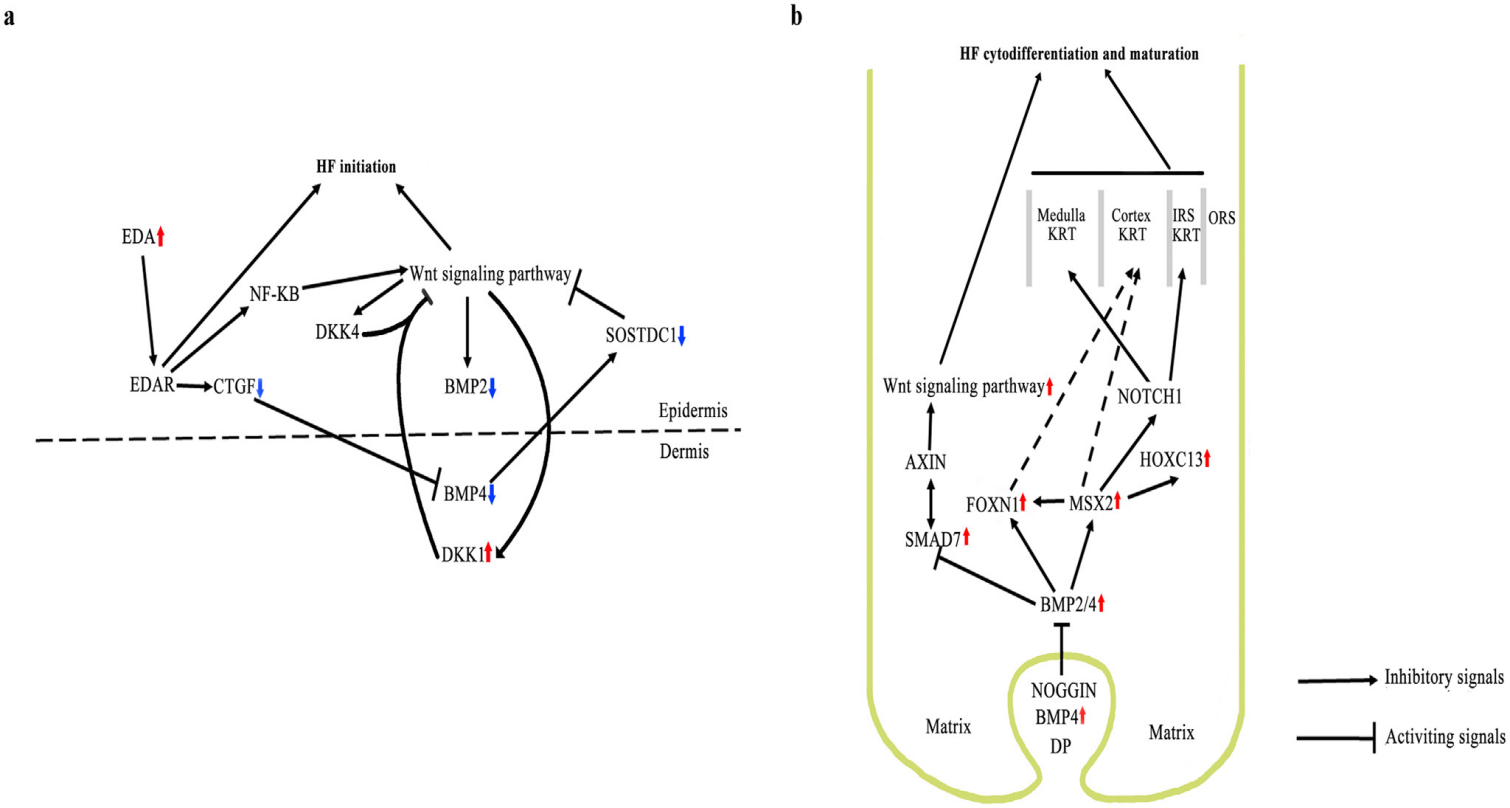
Illustration of HF initiation and cytodifferentiation. (a) The pathway and regulators during the development stages of HF initiation. (b) The pathway and regulators during development stages of HF cytodifferentiation and maturation. Arrows indicate either increased or decreased gene expression.

Although Wnt and TGF-beta/BMP signalling pathways are all involved in HF formation and differentiation, we assume a mechanism by which HF development is regulated by key players within these pathways (Fig 6). For instance, *BMP2* and *BMP4* are down-regulated, and *DKK1* is up-regulated during HF initiation.

In conclusion, our findings have greatly expanded the understanding of transcriptional responses within the three distinct developmental stages of HF. Novel expression patterns for thousands of genes were successfully established during HF morphogenesis. Furthermore, we hypothesise that some DEGs in the three signal transduction pathways (Wnt and TGF-beta/BMP) are independently involved in the cytodifferentiation and maturation of HF.

## Supporting Information

S1 Fig Classification of total raw reads at different developmental stages. After filtering the adaptor sequences, regions containing N sequences and low quality sequences, the nine RNA-seq libraries generated over 20.6 million clean reads in each library. The percentage of clean reads among the raw reads reached 97.52% and 97.86% in each library.
(TIF)

S2 Fig Percent of coverage representing the percentage of a gene covered by reads at each stage. The distribution of distinct reads over different read abundance categories showed similar patterns for all nine RNA-seq libraries. The similarity distribution showed a comparable pattern with approximately 40% of the sequences having a similarity of 80% from the three biological replicates.
(TIF)

S3 Fig Correlations between biological replicates and skin samples. The x- and y-axis correspond to the RPKM value of each sample. The correlation coefficient (r^2^) between two individuals within each group was calculated based on the RPKM value of each individual. Correlation values of two biological replicates at each stage were up to 0.90.
(TIF)

S4 Fig Coefficient analysis of fold change data between qPCR and RNA-seq. Eight genes were selected for qPCR. Data indicating relative transcript level from qPCR and RPKMs from RNA-Seq are means of three replicates in each group. Scatterplots were generated by the log2expression ratios from RNA-seq (X-axis) and qPCR (Y-axis).
(TIF)

S5 Fig Patterns of gene expression by STEM across three phases. Each profile represents an expression pattern. Patterns with colours indicate genes were significantly enriched in this pattern, while blank ones represent non-significance. The number of genes belonging to each pattern is labeled above the profile.
(TIF)

S6 Fig Hierarchical cluster analysis of gene expression profiles from nine skin samples with 50 DEGs. Columns are clustered by libraries and rows are clustered by genes. Dendrogram height indicates distances between clusters in gene expression profiles. Orange indicates up-regulation and blue indicates down-regulation. There were clusters with relatively minor differences for E120 vs. NB. The bottom of each column indicates three replicates in each HF development stage, from left to right, starting with E60_1, E60_2, E60_3, E120_1, E120_2, E120_3, NB_1, NB_2, and NB_3.
(TIF)

S1 Table List of primers for qPCR. (XLSX)

S2 Table Summary of read numbers based on the RNA-Seq data from cashmere goat HF development. (XLSX)

S3 Table Gene with different expression in E60 vs. E120 and E60 vs. NB. (XLSX)

S4 Table List of KEGG pathways for DEGs between different developmental stages. (XLSX)

S5 Table List of genes with different expression in eight profiles. (XLSX)
