# Supplementary figures and images for: Comparative Transcriptome Analysis of Fetal Skin Reveals Key Genes Related to Hair Follicle Morphogenesis in Cashmere Goats
Source: PLoS One. 2016 Mar 9;11(3):e0151118. doi: 10.1371/journal.pone.0151118 (PMC4784850; doi:10.1371/journal.pone.0151118)

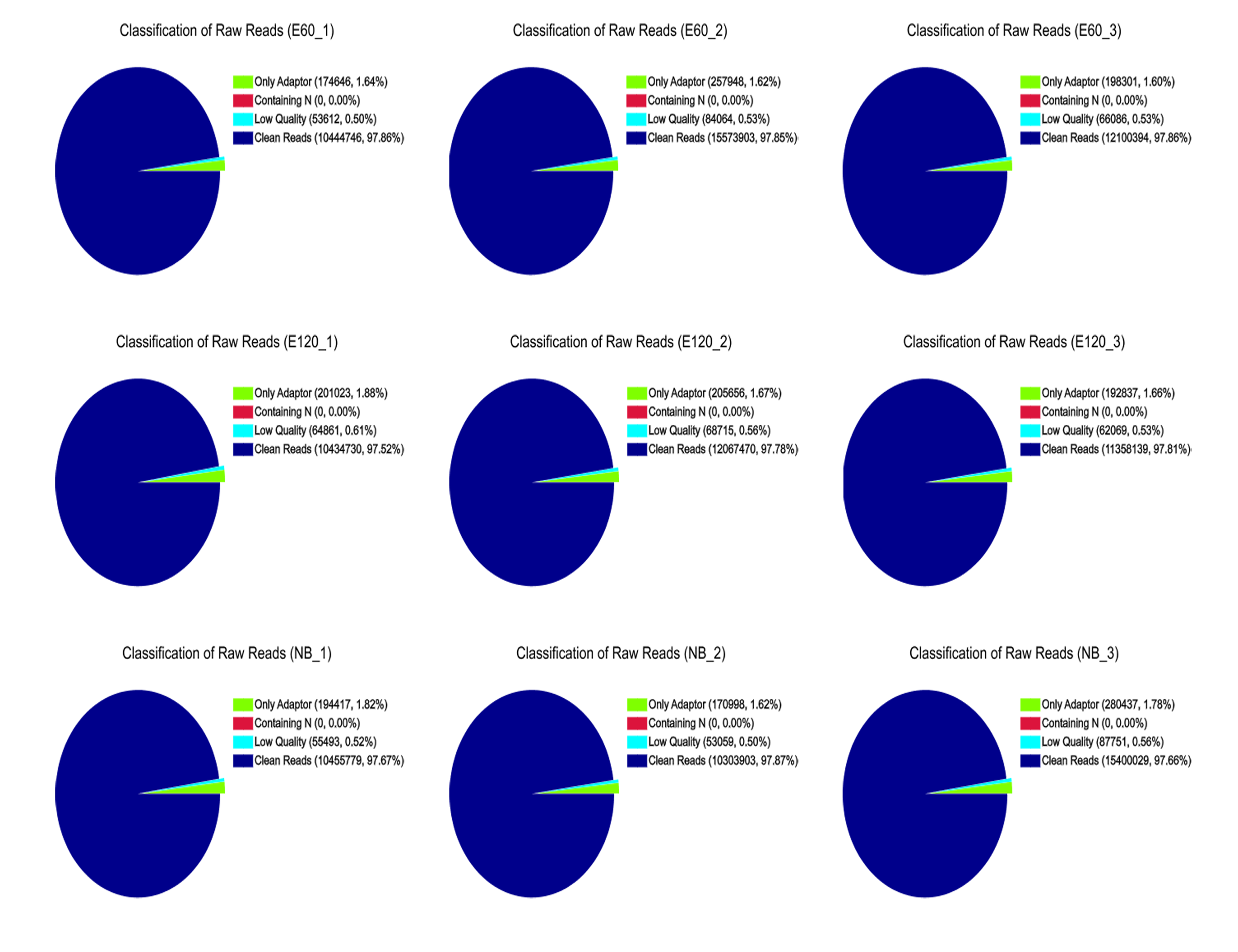

Supplement: S1 Fig — After filtering the adaptor sequences, regions containing N sequences and low quality sequences, the nine RNA-seq libraries generated over 20.6 million clean reads in each library. The percentage of clean reads among the raw reads reached 97.52% and 97.86% in each library. (TIF) [file pone.0151118.s001.tif]

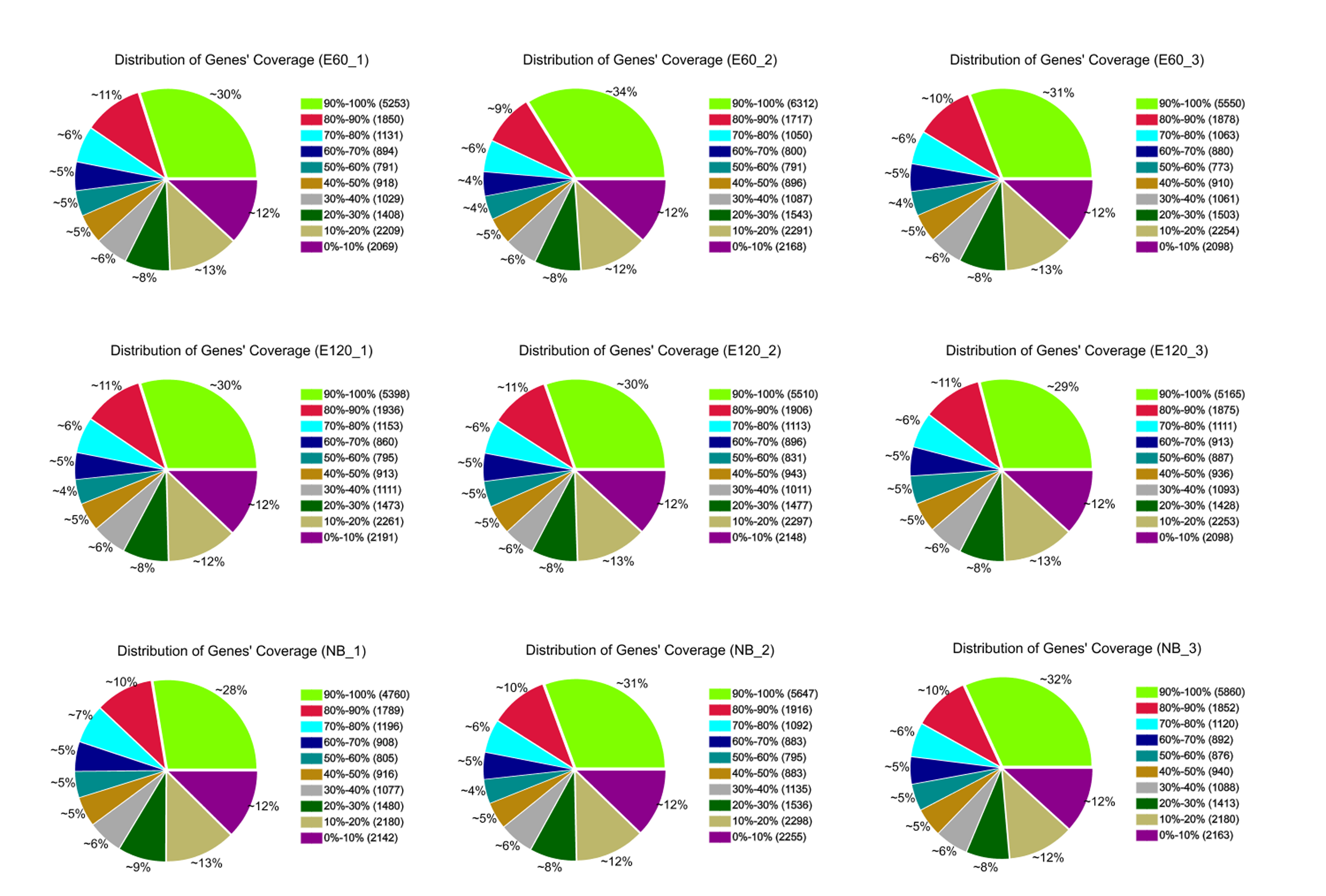

Supplement: S2 Fig — The distribution of distinct reads over different read abundance categories showed similar patterns for all nine RNA-seq libraries. The similarity distribution showed a comparable pattern with approximately 40% of the sequences having a similarity of 80% from the three biological replicates. (TIF) [file pone.0151118.s002.tif]

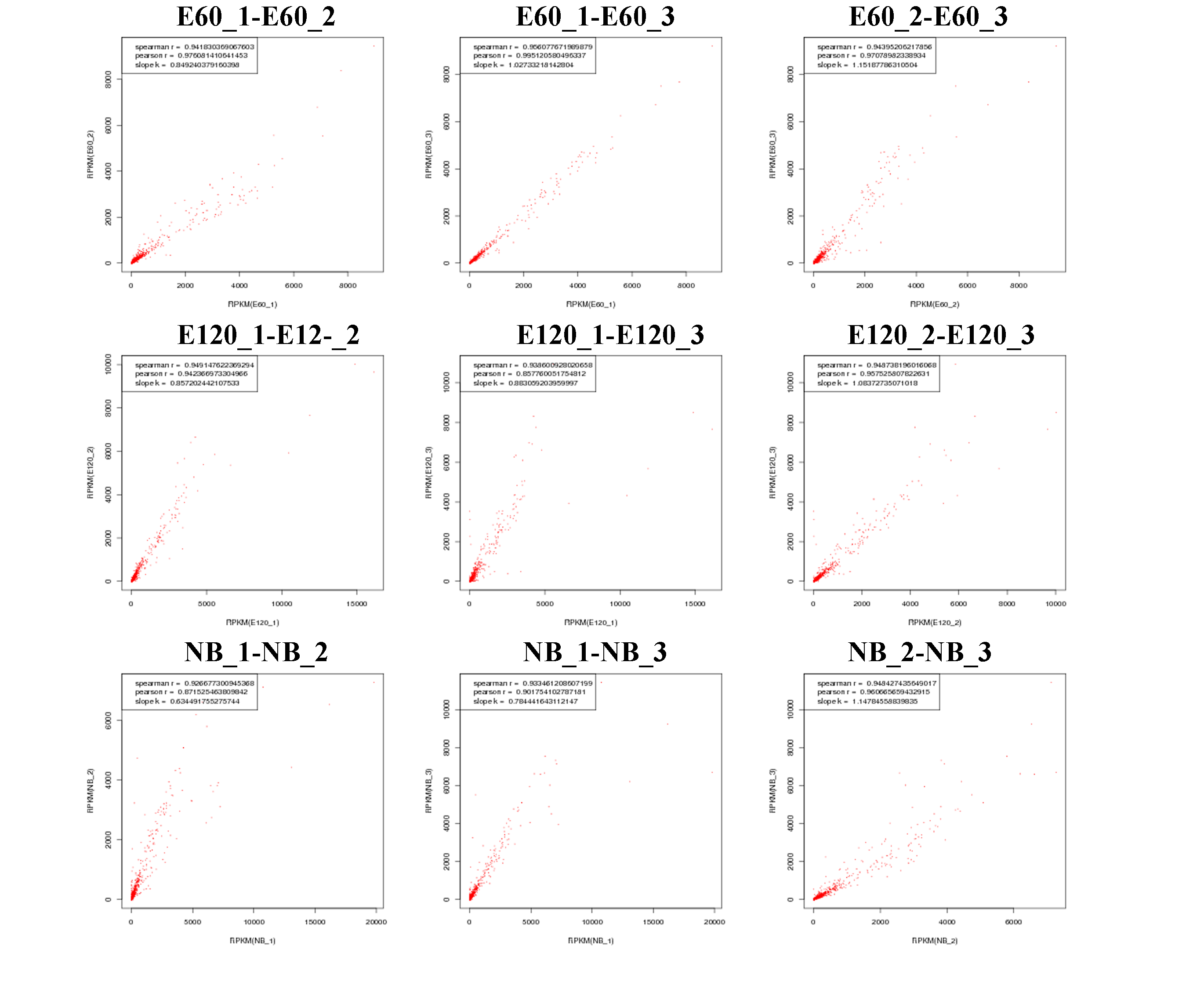

Supplement: S3 Fig — The x- and y-axis correspond to the RPKM value of each sample. The correlation coefficient (r2) between two individuals within each group was calculated based on the RPKM value of each individual. Correlation values of two biological replicates at each stage were up to 0.90. (TIF) [file pone.0151118.s003.tif]

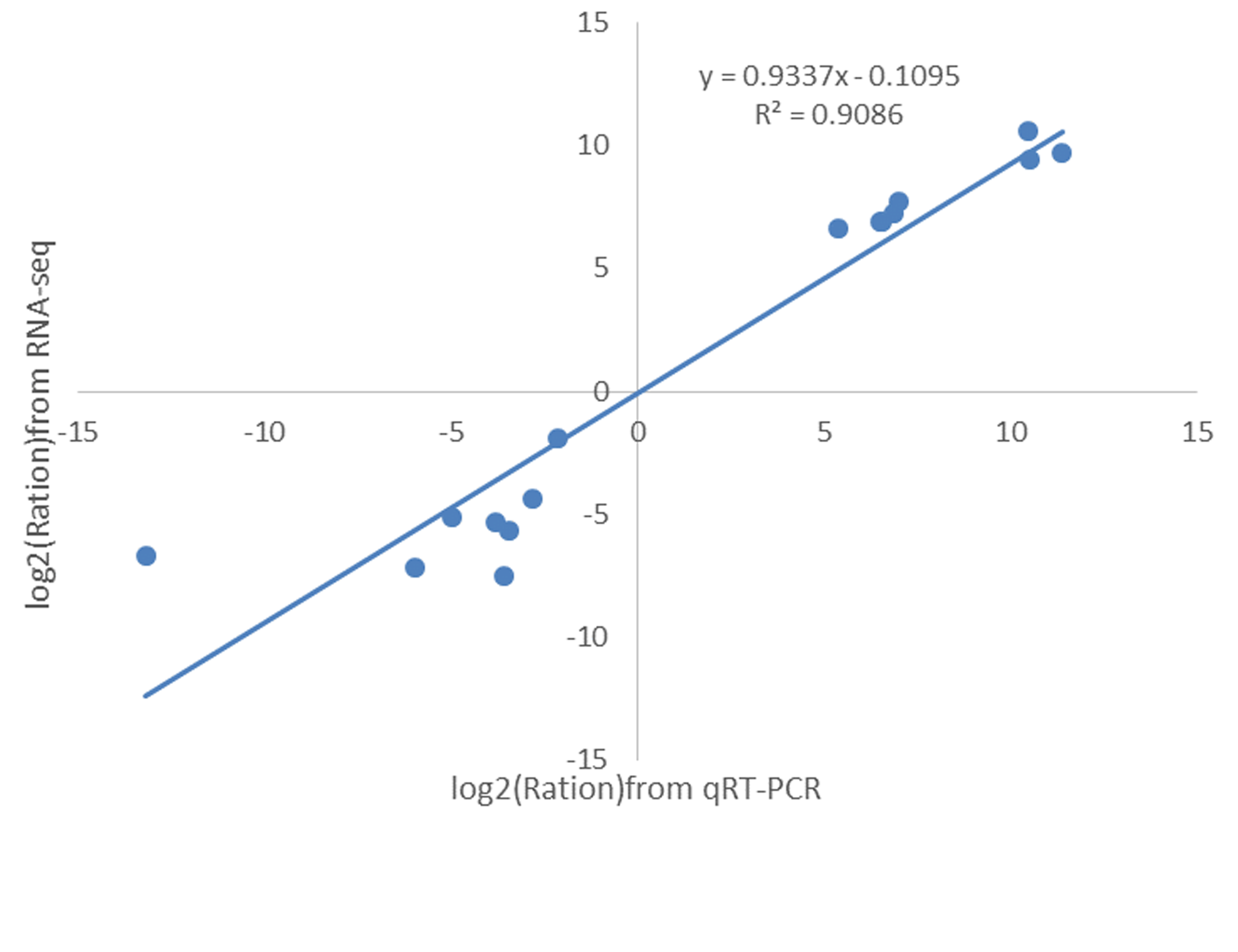

Supplement: S4 Fig — Eight genes were selected for qPCR. Data indicating relative transcript level from qPCR and RPKMs from RNA-Seq are means of three replicates in each group. Scatterplots were generated by the log2expression ratios from RNA-seq (X-axis) and qPCR (Y-axis). (TIF) [file pone.0151118.s004.tif]

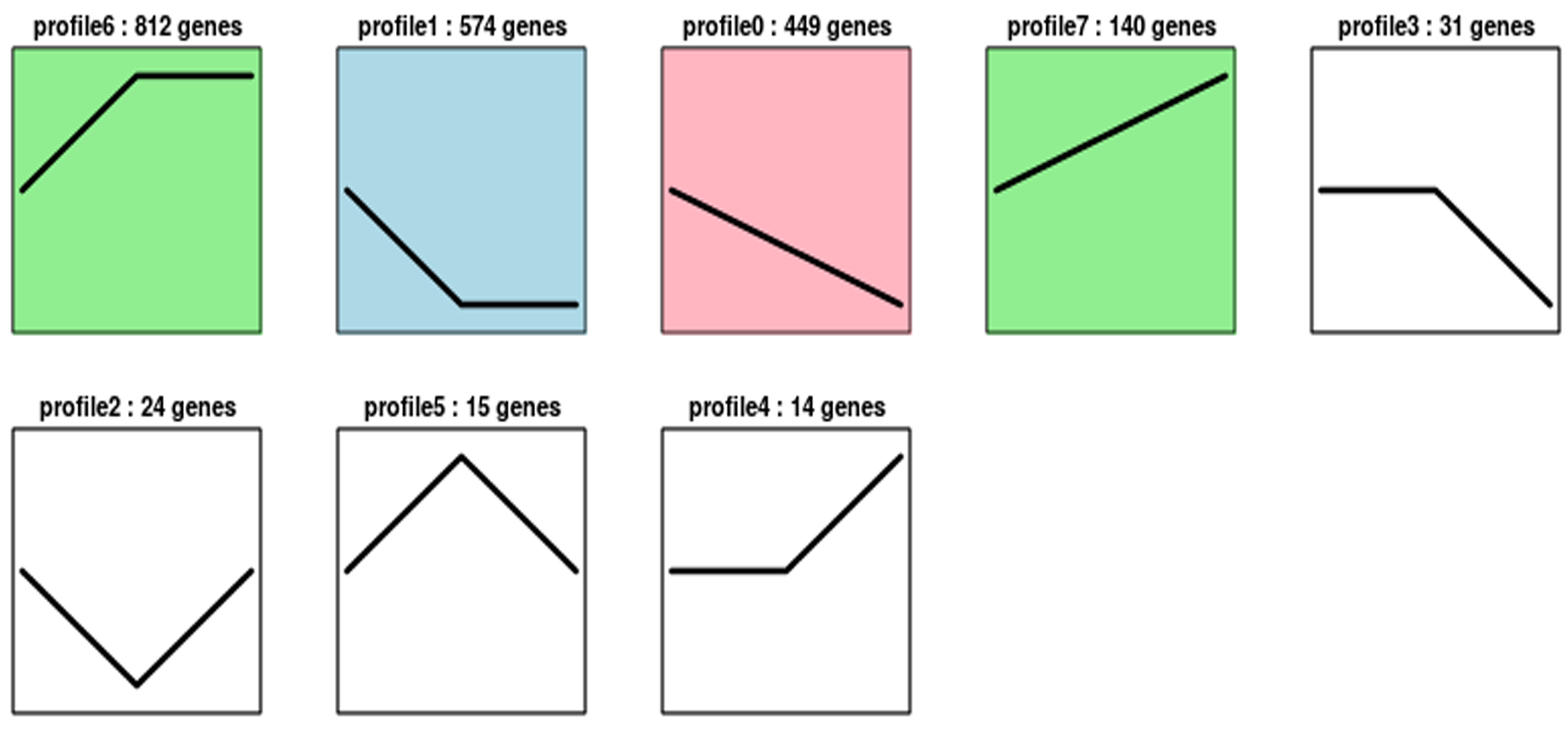

Supplement: S5 Fig — Each profile represents an expression pattern. Patterns with colours indicate genes were significantly enriched in this pattern, while blank ones represent non-significance. The number of genes belonging to each pattern is labeled above the profile. (TIF) [file pone.0151118.s005.tif]

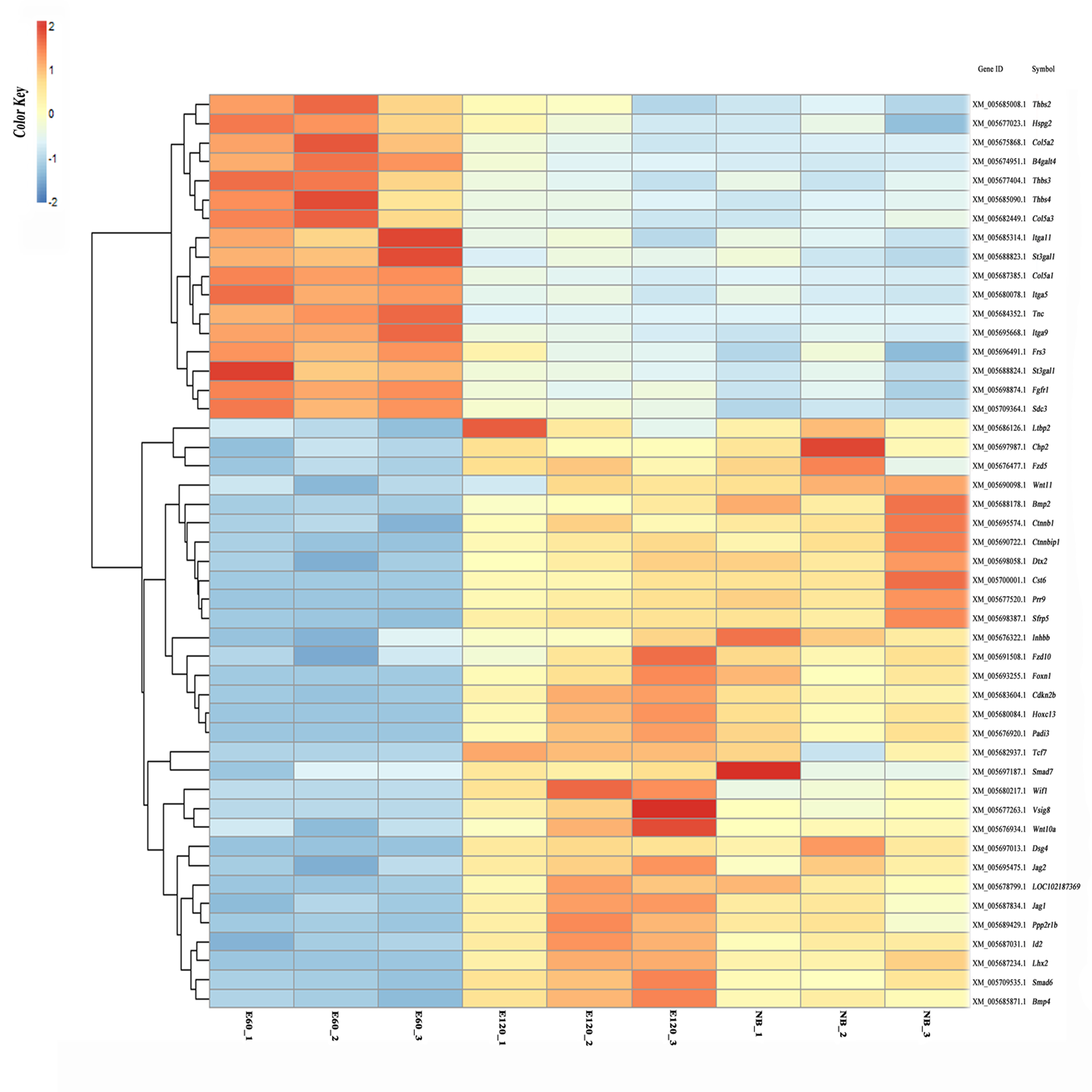

Supplement: S6 Fig — Columns are clustered by libraries and rows are clustered by genes. Dendrogram height indicates distances between clusters in gene expression profiles. Orange indicates up-regulation and blue indicates down-regulation. There were clusters with relatively minor differences for E120 vs. NB. The bottom of each column indicates three replicates in each HF development stage, from left to right, starting with E60_1, E60_2, E60_3, E120_1, E120_2, E120_3, NB_1, NB_2, and NB_3. (TIF) [file pone.0151118.s006.tif]
